# Factors affecting parametrial involvement in cervical cancer patients with tumor size ≤4 cm and selection of low-risk patient group

**DOI:** 10.4274/jtgga.galenos.2020.2020.0153

**Published:** 2021-02-24

**Authors:** Hüseyin Akıllı, Yusuf Aytaç Tohma, Emre Günakan, İrem Küçükyıldız, Mehmet Tunç, Nihan Haberal Reyhan, Ali Ayhan

**Affiliations:** 1 Department of Obstetrics and Gynecology, Başkent University Faculty of Medicine, Ankara, Turkey; 2 Department of Obstetrics and Gynecology, Sivas Cumhuriyet University Faculty of Medicine, Sivas, Turkey; 3 Clinic of Obstetrics and Gynecology, Ankara Keçiören Training and Research Hospital, Ankara, Turkey; 4 Department of Pathology, Başkent University Faculty of Medicine, Ankara, Turkey

**Keywords:** Low-risk cervical cancer, parametrial invasion, less radical surgery

## Abstract

**Objective:**

The primary aim of this study was to evaluate the factors affecting parametrial involvement in cervical cancer patients with tumor size ≤4 cm and selection of the low-risk patient group based on long-term oncologic outcomes.

**Material and Methods:**

Cervical cancer patients operated in the gynecologic oncology division between 2007 and 2013 were retrospectively evaluated. One-hundred and sixty-eight patients with tumor size ≤4 cm were identified. Of these, 159 (86.8%) underwent radical hysterectomy plus pelvic-para-aortic lymphadenectomy and nine (13.2%) underwent fertility-sparing surgery [radical trachelectomy (n=7); large conization (n=2)]. Factors affecting parametrial invasion, including lymphovascular space invasion (LVSI), deep stromal invasion (DSI), lymph node metastases, and tumor size, were evaluated. Statistical analyses were performed using SPSS 23.0 (IBM Corp., Armonk, NY, USA).

**Results:**

Median age was 49.5 years and median tumor size was 2.5 cm (0.45-4 cm). In both univariate and multivariate analyses, the risk of parametrial involvement was increased with LVSI with a hazard ratio (HR) of 3.45 [95% confidence interval (CI): 1.1-10.8] and DSI with a HR of 4.1 (95% CI: 1.18-14.8), while tumor size of ≤2 cm was only significant in univariate analyses. Furthermore, 26 early-stage patients were identified with low-risk factors and they had no parametrial involvement, lymph node metastases, recurrence, or death from disease over 77 months.

**Conclusion:**

Parametrial involvement in low-risk cervical cancer is very rare and less radical procedures may be safe in these patients.

## Introduction

The incidence of cervical cancer and cancer-related deaths have decreased in the last decades due to widespread screening methods (1). Today, in most centers, radical surgery, such as radical hysterectomy or radical trachelectomy, are performed in cases of stage 1a2 and 1b1 cervical cancer as standard care. These radical surgeries may cause sympathetic and parasympathetic nerve damage in the hypogastric plexus and lead to urinary, sexual, and colorectal dysfunction (2). In 2017 Querleu et al. (3) published an updated version of the classification of radical hysterectomy and defined dorsal, ventral and lateral parametrium and how radical parametrectomy can harm branches of  the hypogastric and splanchic nerves.

In stage 1a2 cancers, the risk of recurrence after surgery is 3-5% and 5-year survival is 96-100% (4,5). Some authors suggest that conservative treatment can be applied for these tumors due to the low rate of parametrial and lymphatic involvement (2,4,6,7,8).

Studies have shown that as the extent of stromal invasion increases, lymph node and parametrial involvement rates increase (9,10). Likewise, lymphovascular space invasion (LVSI) and increased tumor size have been associated with parametrial and lymph node involvement. According to these findings, low-risk factors are a tumor size of less than 2 cm, deep stromal invasion (DSI) of less than 50%, and no LVSI (11). In a review published by Ramirez et al. (7) parametrial involvement was reported to be 0.4-0.6% in low-risk patients in retrospective studies. Researchers have focused on the applicability of less radical surgeries, such as simple hysterectomy, simple trachelectomy and wide conization, in these patients (12,13,14,15,16,17).

The primary aim of this study was to identify the factors affecting parametrial involvement in cervical cancer patients with tumor size of ≤4 cm. The secondary aim was to evaluate oncologic outcomes of patients with low-risk factors.

## Material and Methods

This retrospective cohort study was carried out in Ankara, Turkey. The records of cervical cancer patients operated on in the department of gynecology and obstetrics and division of gynecologic oncology between 2007 and 2013 were evaluated. Accordingly, 268 patients were identified, and 68 patients with tumor size of >4 cm were excluded. Also excluded were 20 patients who received neoadjuvant chemotherapy before surgery and 12 patients with microinvasive carcinoma. Rare histologies, such as neuroendocrine tumors were also excluded. Figo stages were 1a2-1b1, according to 2009 system, and some patients were upstaged to stage III according to the revised FIGO 2018 staging system (18). Finally, 168 patients with tumor size of ≤4 cm according to pathology report and who had received no treatment before surgery were enrolled in the study. Tumor histologies were squamous cell, adenocarcinoma and adeno-squamous cell cancer.

Of the enrolled patients, 159 (86.8%) underwent radical hysterectomy (Piver type C1 C2) plus pelvic para-aortic lymphadenectomy and nine (13.2%) underwent fertility-sparing surgery [radical abdominal trachelectomy (n=7); large conization (n=2)]. Lymphadenectomy defined as removal of at least 10 lymph nodes from each pelvic region and five from the para-aortic region.

All of the cases were discussed by experienced gyneco-pathologists. Measurement of tumor diameters were made macroscopically. Tumors were measured in millimeters in three dimensions. One of the three dimensions was the measurement on a single slide in which the extent of the invasion was the greatest. The depth of invasion was measured from the basement membrane of the epithelium that the tumor was considered to have arisen from to the deepest point of invasion. Features that helped in the recognition of LVSI were as follows: 1) a tumor nest within a space associated with other vascular structures; 2) the presence of an endothelial lining, adherence of the tumor cell group to the side of the space; 3) the contour of the intravascular component matching the contour of the vessel; and 4) the presence of adherent fibrin. Immunohistochemistry was not performed routinely but if necessary, immunohistochemical demonstration of an endothelial cell lining was undertaken. For this the D2-40 antibody, specific for lymphatic endothelium, or CD31 and CD34 antibodies, which recognize both the lymphatic and blood vascular endothelium, were used.

All patients were discussed in the tumor board and adjuvant treatment policies were determined according to post-operative risk factors as defined in international guidelines (19).

Every patients was evaluated every three months in the first two years, every six months for the next three years and annually thereafter for five years. Bimanual vaginal examination were done at each visit, a pap test and thoraco-abdominal computed tomography were performed annually. Recurrences were recorded either by imaging modalities and via fine needle biopsy, if needed, before treatment.

This study was approved by the Başkent University Faculty of Medicine Institutional Review Board (approval number: KA13/252). Due to the retrospective design, informed consent from involved patients was waived.

### Statistical analysis

SPSS 23.0 (IBM Corp., Armonk, NY, USA) was used to perform all statistical analyses. Continuous variables were expressed as medians and ranges, and binary variables were reported as counts and percentages. Pearson’s chi-square test, Fisher’s exact test, and the Mann-Whitney U test were used in univariate analysis. The Cox proportional hazards regression model was used to obtain hazard ratios (HRs) and 95% confidence intervals (CIs). All values of p<0.05 were considered statistically significant.

## Results

The median (range) age of the patients was 49.5 (29-80) years and median follow-up time was 77.6 (11-142) months. Median tumor size was 2.5 cm (0.45-4 cm). Sixty-two patients had tumor size of ≤2 cm (36.9%) and 57 (33.9%) patients had stromal invasion of ≤50% of the cervical stroma. Thirty-one (18.5%) of the patients had parametrial involvement and 49 (29.8%) had lymph node metastases. Patients’ characteristic and percentages of adjuvant treatments are given in Table 1.

In univariate analyses, LVSI, tumor size, DSI, and lymph node metastases were found to have significant effects on parametrial involvement (Table 2). In multivariate analyses, positive LVSI increased the risk of parametrial involvement with an HR of 3.45 (95% CI: 1.1-10.8), while DSI increased it with an HR of 4.1 (95% CI: 1.18-14.8). Lymph node metastases also increased the risk of parametrial involvement with an HR of 3.2 (95% CI: 1.3-7.5).

Of the sixty-two patients with a tumor size ≤2 cm, 28 (45%) of them had positive LVSI while 18 (29%) had DSI.

In subgroup analyses 26 patients were found to be in the low-risk group according to pathology reports (negative LVSI, depth of cervical stromal invasion of ≤50%, and tumor size of ≤2 cm) (Table 3). In this low-risk group, no patients had parametrial involvement or lymph node metastases. Median follow-up time was 77 months and none of these patients received adjuvant treatment, none experienced recurrence and none died during the follow-up period.

## Discussion

In recent years, gynecologic oncologic surgeons have focused on performing less radical surgeries for low-risk patients. Therefore, in this study, we evaluated the factors affecting parametrial involvement in cervical cancer patients with low-risk factors. First, positive LVSI, DSI, and lymph node metastases increased the risk of parametrial involvement in all patients. Second, in subgroup analyses, there were no parametrial involvements or lymph node metastases in any patients in the low-risk patient group and no recurrence or death was observed in the 77 months follow-up of these patients, who did not receive any adjuvant therapy.

One of the important points to be considered while planning the treatment of cervical cancer is determination of the risk group of the patient. Therefore, studies have investigated various factors in order to correctly classify patients. In the study of Covens et al. (20), which included 842 stage 1A1-1B1 patients who underwent radical surgery, there was a parametrial involvement rate of 0.6% in 536 patients with tumor size of less than 2 cm, DSI of <10 mm, and negative lymph nodes. In another study, advanced age, poor histology, large tumor size, DSI, LVSI, vaginal involvement, and pelvic or para-aortic lymph node metastasis were demonstrated as factors predicting the high-risk group (13). When subgroup analysis was performed, the same authors found that for negative LVSI and tumor size of ≤2 cm, the parametrial involvement rate in patients without lymph node metastasis was 0.4% (13). In our study, similar results were obtained, and in subgroup analyses, there ws no parametrial involvement or lymph node metastases in any patient in the low-risk group and no recurrence or death observed in the 6-year follow-up of these patients. All of the preceding retrospective studies and our data show that the parametrial involvement rate in patients within the low-risk group is less than 1%. This should be kept in mind so that more conservative surgeries may be safe and viable treatment options for patient groups with these predictive factors. In our cohort the percentage of parametrial involvement in patients with a tumor size less than 2 cm was 11% and this number is relatively high compared to earlier studies. The main reason for this inconsistency seems to be the high rates of positive LVSI and DSI in this sub-group.

In the literature, there are many studies concerning the oncologic outcome in patients with early-stage cervical cancer who underwent less radical surgeries, such as simple trachelectomy, conization, or simple hysterectomy (21,22,23). In these studies, the oncologic results were satisfactory and, according to these outcomes, gynecologic oncologic surgeons and patients are still expected to shift towards less radical surgery, although many centers continue to perform radical surgeries in this patient group. The most important reason for this is the lack of convincing evidence to the contrary in this area. Although the results of our study contribute to and confirm the previous reports, and we recommend less radical surgery, further prospective studies are still required. Our results should be further supported with the results of ongoing prospective studies, including the SHAPE and CONCERV studies, which are investigating this topic (24,25).

### Study Limitation

The present study has some limitations. The first and main limitation is the retrospective design; as a result, the possibility of selection bias could not be completely excluded. Another limitation is that, although comparable to similar studies in the literature, the number of patients analyzed in the subgroups was relatively low. This particular study shows post-operative risk factors for parametrial involvement but these risk factors should be combined with pre-operative findings, such as magnetic resonance imaging and biopsy histopathological reports.

## Conclusion

At present, the available data suggest that patients with low-risk, early-stage cervical cancer maybe good candidates for conservative surgery so that conization, simple trachelectomy, or simple hysterectomy and pelvic lymphadenectomy may be good options for these patients. For this reason, all patients with cervical cancer should be examined in detail with pre-operative pelvic examination and imaging methods, and less radical surgery options should be considered in patients with low-risk factors following careful assessment.

## Figures and Tables

**Table 1:**
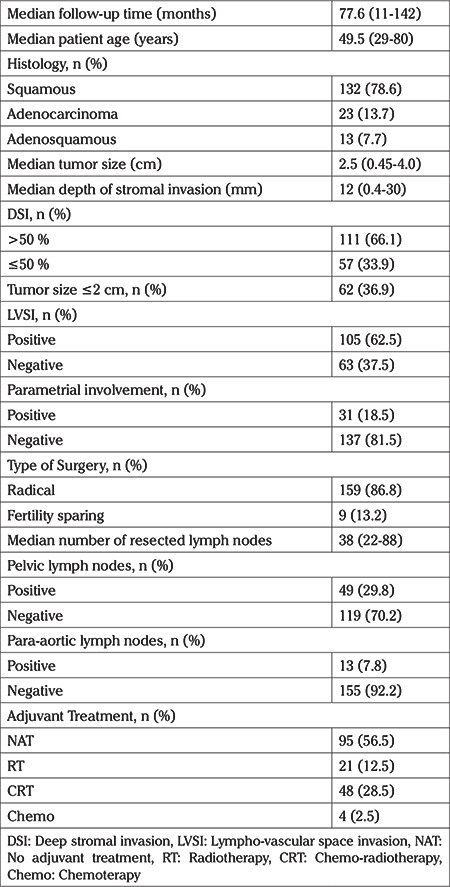
Characteristics of patients

**Table 2:**
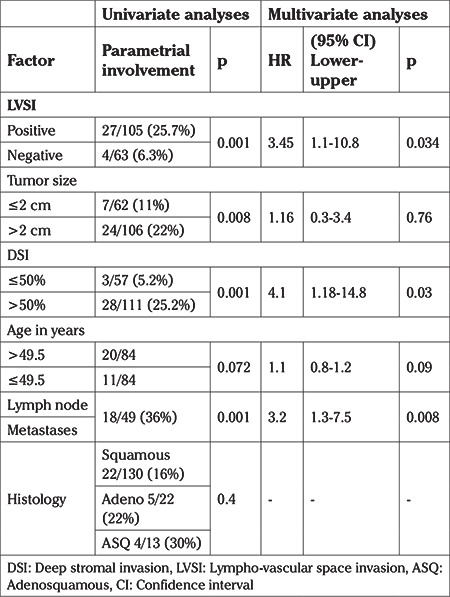
Univariate and multivariate analyses of factors affecting parametrial involvement

**Table 3:**
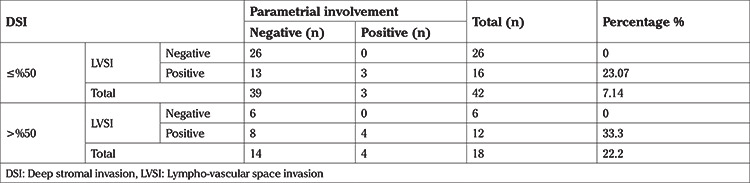
Parametrial involvement rates of patient with tumor size ≤2 cm according to LVSI and DSI
